# Measurement uncertainty of blood ethanol concentration in drink-driving cases in an emergency laboratory

**DOI:** 10.11613/BM.2017.030708

**Published:** 2017-10-15

**Authors:** Yasemin Ustundağ, Kağan Huysal

**Affiliations:** Department of Clinical Biochemistry, Bursa Yuksek Ihtisas Faculty, Saglik Bilimleri University, Bursa, Turkey

**Keywords:** blood ethanol, measurement uncertainty, driving accidents

## Abstract

**Introduction:**

The quality of blood ethanol concentration (BEC) determination is important because of its legal ramifications. Measurement uncertainty provides quantitative information about the quality and reliability of test results. In this study, we aim to calculate the measurement uncertainty for the ethanol test in our laboratory measured with a Synchron Systems Ethanol assay kit by employing an enzymatic rate method on the Beckman-Coulter Olympus AU400 auto analyzer (Beckman Coulter Inc, Melville, USA).

**Materials and methods:**

The measurement uncertainty values were calculated in accordance to the Nordtest guidelines. All vehicle drivers involved in a traffic accident were retrospectively inspected for the BEC test conducted during July to December 2016 in our emergency laboratory.

**Results:**

A 1034 vehicle drivers had their BEC tested. The results for 181 drivers were > 0.50 g/L and reported as positive. The serum ethanol concentration in those showing a positive result was 2.04 ± 1.01 g/L, over four times the legal limit. The median BEC in those showing a negative result was 0.03 (IQR: 0.03) g/L. The expanded uncertainty obtained was 19.74%. When measurement uncertainty values were added to the results of the 1034 drivers who were retrospectively screened, eight vehicle drivers had results with 95% confidence intervals that exceeded the legal limit 0.50 g/L.

**Conclusions:**

The BEC test results for vehicle drivers with values close to legal limits should be reported as the obtained ethanol concentration with corresponding measurement uncertainty.

## Introduction

According to estimates of the World Health Organization, traffic accidents were the ninth leading cause of death in 2004, and could become the fifth by 2030 (*1*). Ethanol abuse has been identified as one of the most significant risk factors for traffic accidents in several countries, including Turkey (*2*-*4*). The extent to which an individual may be under the influence of ethanol is usually determined by either measuring the ethanol content in the breath or the ethanol in the blood.

Turkish road traffic law enforces a blood ethanol concentration (BEC) limit of 0.50 g/L for private vehicle drivers. However, it is reduced to zero for those carrying people on public or commercial transport (according to Road Traffic Act # 2918 dated 18th July 1997 in Turkey) (*4*). The emergency department laboratory of our hospital provides service to police officers in order to measure the BEC of intoxicated drivers involved in car accidents and those suspected of ethanol intoxication.

Blood ethanol concentration, which also has forensic significance, is reported as either positive or negative with regard to legal limits. When presenting and interpreting results, the quality of analytical data is of utmost importance because of the legal ramifications of forensic reports. Therefore, it is particularly important to correctly interpret the laboratory’s BEC results that are close to the legal limits (*4*).

In principle, it is assumed that no measurement is accurate and that the actual value of an analytical measurement is subject to uncertainties in the measurements. Measurement uncertainty is defined by the International Organization for Standardization (ISO) 15189 as “a parameter associated with the result of a measurement that characterizes the dispersion of values.” The value of measurement uncertainty significantly contributes to the evaluation of test results in clinical practice. If measurement uncertainty is given along with the result, the end user will be able to evaluate what the real value represents (*5*).

In this study, we aimed to calculate the measurement uncertainty for BEC test in our laboratory and to re-evaluate the vehicle drivers’ results with respect to the calculated uncertainty value.

## Materials and methods

### Laboratory ethanol analysis

Approval was obtained for this study from the Regional Ethics Committee of Uludag University, Bursa, Turkey (2017-11/34). A retrospective descriptive study was carried out at the Bursa Yuksek Ihtisas Education and Research Hospital.

The blood collection and handling process was standardized as recommended by the Clinical and Laboratory Standards Institute (CLSI) (*6*). Briefly, the routine method for BEC determination involves simultaneously obtaining two tubes of venous blood immediately after the arrival of vehicle drivers. Benzalkonium chloride is used for cleansing the venipuncture site. Paired samples were drawn in plain blood collection tubes (Vacusera, Disera A.S. Izmir, Turkey). Time of collection is recorded in the data management system. Samples were hand-delivered directly to the emergency laboratory by nurses and then centrifuged at 3000xg for 10 minutes to separate serum, no longer than 30 minutes after collection. Measurements were done in both specimens immediately upon centrifugation.

Blood ethanol concentration was analyzed using a Synchron Systems Ethanol Assay kit (A-E 474947) by employing an enzymatic rate method on the Beckman–Coulter Olympus AU400 auto analyzer (Beckman Coulter Inc, Melville, USA). In this reaction, alcohol dehydrogenase catalyzes the reaction of ethanol and nicotinamide adenine dinucleotide (NAD) to acetaldehyde and NADH. The rate of change in absorbance at 340 nm is used to determine the ethanol concentration in the sample. Synchron Systems Ethanol Assay kits’ information sheet reports an analytical measurement range of 0.05 to 6.00 g/L, with a lower limit of quantification of 0.04 g/L and precision between 1.3% and 2.6%.

For ethanol analysis, our laboratory participates in an external quality control program called the Bio-Dev Clinical Laboratory Evaluation Programme (Bio Development s.r.l.; Milano, Italia). In this programme, the matrix of the external quality control samples was serum, and targets were set as peer group mean. The number of participants using the external quality control program for ethanol during the study period was 1011, and the number using the same laboratory method as ours was 56.

In our study, the measurement uncertainty values were calculated, and the laboratory data were inspected retrospectively for the BEC test conducted during the period from July to December 2016 in the emergency laboratory. Cases were taken from medical documents collected in the emergency laboratory consisting of all vehicle drivers involved in a traffic accident and referred by police officers for suspicious ethanol intoxication. Laboratory data were extracted from hospitals’ electronic records including patient demographics, specimen collection and report date and time as part of the routine data management system.

### Estimation of measurement uncertainty

The measurement uncertainty values were calculated in accordance to the Nordtest guidelines (*7*). According to the Nordtest approach, the combined measurement uncertainty is calculated by using the within-laboratory reproducibility (intermediate precision) *s*_Rw_ and the uncertainty due to possible laboratory bias *u(bias)*. Both can be conveniently estimated from existing internal and external quality control data, thereby making uncertainty estimation easier for routine laboratories (*8*).

Internal quality control results studied between July and December 2016 were collected at normal and pathological ethanol concentrations (control set low/high, Lot 601471, 601472 Synchron, Beckman Coulter Inc., Melville, USA). The actual concentrations of the internal quality control materials were 0.42 g/L to 0.60 g/L for level 1, and 0.86 g/L to 1.16 g/L for level 2. Internal quality control materials (N = 368) were analyzed as samples; and were included in the calculation of the mean and standard deviation (SD).

Relative standard deviation (RSD) values were calculated (*i.e.* RSD_Normal_ and RSD_Patological_). Because we have more than one set of control data, the uncertainty was calculated using both control values with the formula:


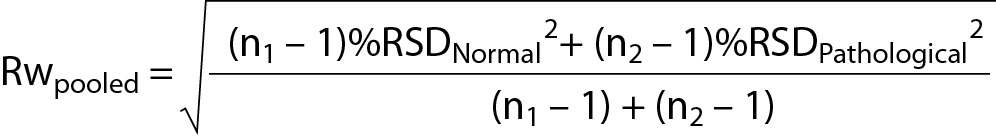


### Bias calculation

Bias is the percent of systematic difference (positive or negative) between measurement results and the accepted true value (*9*). The easiest and most convenient method for bias estimation in routine clinical laboratories is to use existing external quality control data based on the peer group mean (*8*).

We calculated the combined bias using the formula:


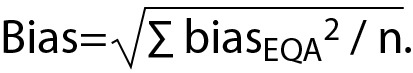


For ethanol analysis, a bias of ± 10% or better is expected (*9*).

Standard uncertainties were calculated by taking the square root of the sum of the squares, as follows:


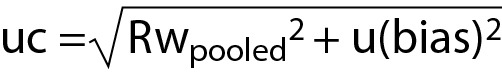


The combined standard uncertainty is multiplied by 2, *i.e.*, *U =* 2*u_c_*, to give an expanded uncertainty with a confidence level of 95%. Allowable total error according to the Clinical Laboratory Improvement Amendments (CLIA 88) is ± 25%.

### Statistical analysis

Data were evaluated using the IBM Statistical Package for Social Science software program (SPSS for Windows, Version 21.0, SPSS Inc. Chicago, USA). The normality of the continuous variables was tested with the Kolmogorov-Smirnov test. Data were expressed as mean ± standard deviation (SDs) when normally distributed or median (interquartile range; IQR) otherwise.

## Results

We analyzed two sets of quality control samples during the study period. Their laboratory means and SDs were 0.52 ± 0.04 g/L and 1.03 ± 0.04 g/L, respectively. The calculated Rw_pooled_ using the internal quality control data was 5.56%. The bias obtained from the external quality control programme was 2.53%, - 2.27%, 8.00%, and 13.82%, and calculated bias as 8.16%. The standard uncertainty calculated from the uncertainty components was 9.87%, and the expanded uncertainty was 19.74%.

**Figure 1:**
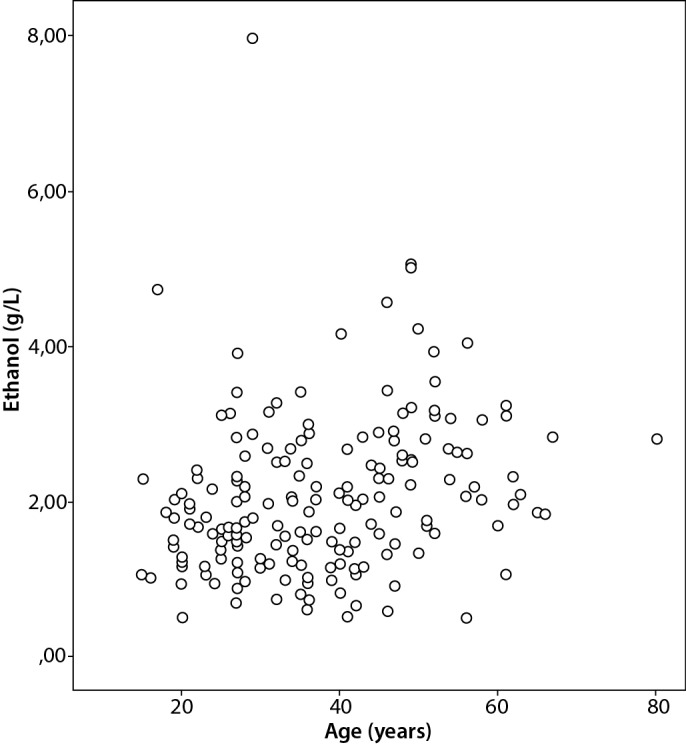
Age distribution of blood ethanol concentrations in intoxicated vehicle drives (N = 181)

**Figure 2:**
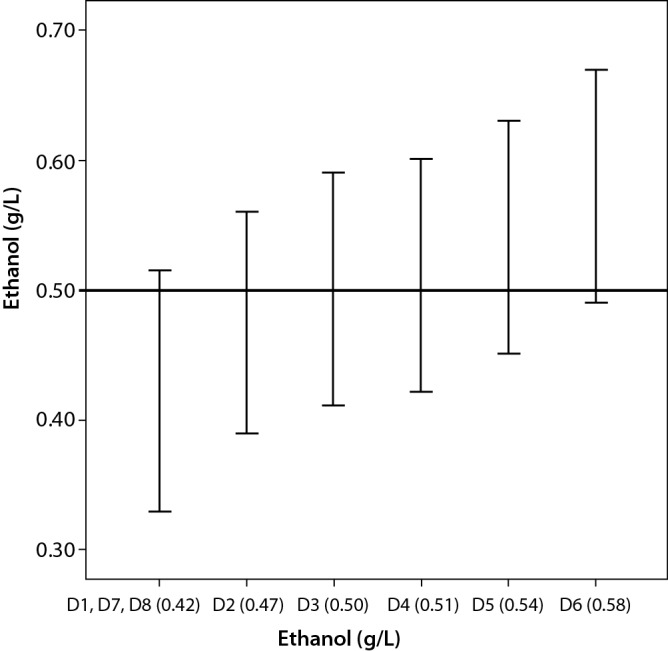
Ethanol results for eight vehicle drivers (D) with results out of 95% confidence intervals for uncertainty at the legal limit. Legal limit is 0.50 g/L (bold). Horizontal bars represent the range of ethanol concentrations (min-max). Equal ethanol concentrations were found for D1, D7 and D8.

During July to December 2016, 1034 vehicle drivers’ BEC were tested. Of these, 978 (94.3%) were male. The results for 181 of the 1034 patients were > 0.50 g/L and reported as positive. Positivity rates were 7.8% for ethanol. Of those who tested positive, 171 (94.4%) were male. Figure 1 shows the distribution of positive cases by age. The average BEC in those with positive result was 2.04 ± 1.01 g/L, which is over four times the legal limit. The median BEC in those showing a negative result was 0.03 (IQR: 0.03) g/L. When measurement uncertainty was factored into the interpretation of the results, eight vehicle drivers had results with 95% confidence intervals that fell on either side of the decision limit 0.50 g/L (Figure 2). Measurement uncertainty values were 19.74% for ethanol, defined as ± 0.09 g/L at 0.5 g/L.

## Discussion

We present the results for the determination of ethanol in blood and the estimation of the uncertainty of these measurements. We calculated the measurement uncertainty values for the ethanol being studied in our laboratory. In our study, the measurement uncertainty of ethanol was 19.74%. As a result, the BEC positivity cut off level is reported as 0.50 ± 0.09 g/L. Our uncertainty value (19.74%) was lower than the acceptable values according to CLIA’88 (*i.e.* blood alcohol concentration target value ± 25%) (*10*).

There are a limited number of studies which reported measurement uncertainty data specifically targeting BEC test (*11*-*14*). Similar to our study, Ince *et al.* calculated the measurement uncertainty of ethanol that was measured using the enzymatic method (MULTIGENT Ethanol Reagent, Abbott Diagnostic, Germany) on Architect c8000 analyzers (Abbott Diagnostic, Germany). At threshold level (0.50 g/L) they reported measurement uncertainty results of ± 0.04 g/L (*11*). However, they used calibrator uncertainty and stability uncertainty instead of external quality control test results.

Using data from external quality assessment schemes in monitoring the measurement uncertainty of blood ethanol test is a novel and practical approach (*8*).

In our study, the bias of external quality control data, which contributes to measurement uncertainty, was high. It is accepted that an external quality control program is a good mean of detecting the systematic error of a parameter because only the uncertainty of handling the control sample, and the analytical uncertainty are included (*15*). The major weakness of using the external quality control for bias estimation is the number of laboratories participating in the external quality program, problems during the transport of external quality control materials, improper dilution during the preparation of stabilized liquid control serum, and misreporting during analysis. On the other hand, an external quality control program allows assessment of the results nationally or even internationally. When there are enough participants; group mean values are considered to be close to those obtained from the reference laboratory (*16*, *17*).

The positivity rates for ethanol measured in our laboratory between July and December 2016 for 1034 samples are 7.8%. In the current practice, the result measured is assessed according to whether the decision value is exceeded or not. However, if the results are evaluated together with the uncertainty values, results close to the decision value will be affected. For example, a result reported above the decision value of 0.51 g/L may be any value between 0.42 and 0.60 g/L. Since ethanol results given above or below certain thresholds may result in judicial decisions about the person concerned, the knowledge of measurement uncertainty values for BEC is more important when compared to other routine laboratory tests. During the study period, most vehicle drivers admitted to our trauma center had BECs well over the legal amount allowed. Eight vehicle drivers had results with 95% confidence intervals that fell on either side of the legal limit of 0.50 g/L. For these drivers, the true results may be above or below the permissible legal limits and should be interpreted with caution. After the calculation of the measurement uncertainty values, we suggest that most of the test results can be reported.

In lawsuits filed against drivers, the method of measurement should be validated and confirmation with a sensitive method is needed to prevent erroneous interpretations. Various laboratory tests can be used for the determination of ethanol in serum. Among these, the enzymatic or gas chromatography mass spectrometry (GC-MS) are the most commonly used (*11*-*14*). The latest generation of headspace GC-MS techniques are superior with lower measurement uncertainty values (*13*, *14*).

Sklerov *et al.* calculated the measurement uncertainty for blood ethanol testing using headspace GC-MS including as many sources of variability (operators, instruments, environmental conditions, *etc.*). Long term precision calculated from internal control values yield a RSD of 0.00187 g/L. Also their relative, combined standard uncertainty was ± 2.7% (*14*).

Nevertheless, GC-MS methods are generally not well suited for routine emergency clinical laboratories due to the manual nature of the methods.

In our study, the median BEC in those showing a negative result was 0.03 g/L.

Zero-ethanol limit detection for commercial and official vehicle drivers in traffic is challenging for clinical laboratories and needs more sensitive and reliable determination methods to avoid misinterpretations. The detection limits of the method should be known, so that analytical reports contain less-than values rather than reporting a zero. In the Synchron Systems Ethanol Assay kit, the lowest measurable concentration that can be distinguished from zero with 95% confidence is reported 0.04 g/L by the manufacturer. Accordingly, for the advanced and sensitive HS-GS method zero-ethanol cut-off level is suggested to be set at BEC < 0.01 g/L (*18*).

However, besides analytical challenges when evaluating the zero alcohol limit, endogenous ethanol production which does not originate but is related to spontaneous production by different metabolic pathways and patients suffering from various metabolic disorders (*e.g.,* diabetes, cirrhosis) should also be considered (*19*, *20*). In a large study of alcohol-free healthy individuals, mean BEC of 0.01 ± 0.04 g/L and a maximum blood ethanol concentration 0.15 g/L was reported using sensitive headspace GS/MS (*20*).

The major limitation of our study is that measurement uncertainty is calculated for the analytical process, not taking into account the preanalytical and postanalytical phases.

In conclusion, when starting to use a standard test method for ethanol measurement, measurement uncertainty should be estimated, since it may lead to inaccurate analyses served before the courts. BEC test results close to the legal limits should be reported with a confidence interval containing the true ethanol concentration with its 95% confidence interval.
